# Power Consumption and Rubber Phase Evolution in an Intermeshing Mixer: A Three-Dimensional Non-Newtonian Volume-of-Fluid Computational Fluid Dynamics Analysis

**DOI:** 10.3390/polym18101163

**Published:** 2026-05-09

**Authors:** Fareed Konadu Osman, Dandan Hou, Lei Han, Qi Zhou, Jie Gao, Chunsheng Zhang, Leilei Miao, Alfredo Iranzo

**Affiliations:** 1 School of Energy Science and Engineering, Harbin Institute of Technology, Harbin 150001, China; 2 Zhongce Rubber Group Company Limited, Hangzhou 310018, China; 3 Dalian Rubber and Plastics Machinery Company Limited, Dalian 116036, China; 4 Energy Engineering Department, School of Engineering, University of Sevilla, 41092 Sevilla, Spain

**Keywords:** rubber mixing, intermeshing rotor mixer, dynamic remeshing, non-Newtonian flow, power consumption

## Abstract

This study investigates the influence of key operating parameters of fill factor, rotor speed, and rotor wear on the power consumption of an isothermal intermeshing internal mixer. A three-dimensional computational fluid dynamics (CFD) model incorporating dynamic remeshing was developed using the finite volume method to solve the continuity and momentum equations for non-Newtonian rubber flow. The dynamic remeshing approach enabled accurate tracking of the moving rotor geometry and maintained mesh quality under varying operating conditions. The model integrates the actual mixer geometry and rheological properties of the rubber, and was validated against plant-scale power consumption data, showing good agreement. Simulations were performed across a range of operating conditions to quantify the effects of each parameter. Results indicate that increasing the fill factor from 50% to 82% raises normalized power from 14–19 kW/% to 17–22 kW/%, with higher levels producing extensive shear stress coverage to the rotor barrels but at the cost of potential clogging and reduced energy efficiency. Increasing rotor speed from 35 to 60 rpm increases normalized power from 20–22 kW/rpm to 22–23 kW/rpm, as higher rotor speeds intensify the local shear stress and strain rate fields near the rotor tips, thereby increasing power consumption. Rotor wear was found to significantly influence power consumption, with increasing wear leading to a progressive reduction in energy demand. The results indicate that worn rotor conditions reduce mechanical energy transfer due to diminished rotor–material interaction and increased clearances, resulting in lower shear stress generation within the mixing chamber. These findings identify operational windows that minimize energy costs while maintaining effective wall shear stress, offering practical guidance for optimizing mixer performance.

## 1. Introduction

Intermeshing rotors are widely used in rubber mixing because their counter-rotating geometry generates high local shear rates and extensional deformation. This causes repeated material reorientation in the inter-rotor and rotor wall regions. The narrow regions between the rotors and the chamber walls are critical for dispersive and distributive mixing of highly viscous rubber compounds [1]. While their primary advantage lies in effective material mixing, these machines are also significant consumers of energy, and power demand is a major operational cost in industrial rubber mixing [2,3,4]. Despite their widespread industrial use, no prior study, to the authors’ knowledge, has systematically quantified the effects of fill factor, rotor speed, and rotor wear on power consumption in intermeshing rotor rubber mixers. At present, several scholars have conducted studies using both numerical simulations and experimental techniques in an effort to understand the internal flow mechanisms of rubber mixing. Early efforts often utilized simplified two-dimensional (2D) models to capture the primary flow features while minimizing computational cost. Liu et al. [5] analyzed the transient distribution of rubber in a mixer using a 2D model. Han and He [6] employed a three-dimensional model to study differences between partial and complete filling under isothermal conditions. Their results show similar flow distribution patterns. Poudyal et al. [7] and Ahmed et al. [8] also used a 2D model to compare isothermal and non-isothermal mixing. They concluded that the evolution of mixing evaluation indices was consistent under both thermal conditions. However, the reduced dimensionality in these models inherently limits their ability to capture the complex three-dimensional flow structures and rotor–material interactions that occur in practice. The development of computational 3D modeling has provided a more complete view of internal mixer behavior, allowing detailed assessment of geometry and operating parameters. Connelly and Kokini [9] were pioneers of this method, where they applied 3D modeling to investigate rotor mixing effects in sigma-type structures. Wang et al. [10] compared 2D and 3D computational models for an internal tangential mixer at varying rotational speeds. They illustrate the importance of fully three-dimensional simulations for capturing realistic flow fields.

Several 3D numerical studies have been conducted under various operating conditions to determine the influence of dispersive and distributive behaviors of an internal mixer. M. Falahati [11] conducted an analysis on a Banbury mixer where he concluded that fill factor strongly governs rubber-phase distribution and mixing efficiency. Ahmed et al. [8,12] also conducted comparative numerical studies and concluded that best fill factors were between 70 and 80% for a two-wing non-intermeshing mixer. More studies on tangential rotor mixers were conducted to determine the influence of some operational characteristics such as speed, speed ratios and fill factor [13,14], all in a bid to further understand the underlying mechanisms of rubber mixing. While these studies have significantly advanced the understanding of mixing efficiency and flow behavior, their primary focus has remained on dispersive and distributive mixing metrics, with comparatively limited attention given to the direct quantification of power consumption, which is a key parameter in industrial mixer operations. Furthermore, most existing studies consider fixed rotor geometries, and do not account for geometry degradation effects such as rotor wear, despite its practical importance in long-term mixer performance [15,16,17]. In addition, although three-dimensional modeling has improved the representation of internal flow structures, the combined treatment of non-Newtonian rheology, free-surface evolution, and dynamically changing rotor geometry under partial-fill conditions remains computationally challenging and has not been systematically applied to evaluate energy demand in intermeshing rotor systems.

A major challenge underlying these limitations lies in the dynamic remeshing requirements associated with numerical simulations of intermeshing mixers. Rubber mixing involves the motion of rotors in close proximity, requiring continuous mesh adaptation to prevent element distortion and maintain numerical accuracy. Achieving this remeshing dynamically, without introducing excessive numerical errors, is computationally demanding and has posed significant challenges for a high-fidelity simulation of such systems.

To address these challenges, the present study focuses on the direct quantification of power consumption in an intermeshing internal mixer under varying operating conditions. Accurate prediction of power demand requires high-fidelity simulations that incorporate realistic rotor geometry, non-Newtonian material behavior, and moving boundaries associated with partial filling. Dynamic remeshing is therefore employed to resolve the close-clearance motion of the rotors without mesh distortion. In addition, the influence of rotor wear, which alters the effective flow domain and shear characteristics, is systematically examined to provide further insight into its impact on energy demand.

This study provides a quantitative framework for linking operating conditions and rotor geometry to power consumption in intermeshing internal mixers, offering insight into energy demand under industrially relevant conditions.

## 2. Materials and Methods

### 2.1. Geometry Description

**Figure 1:**
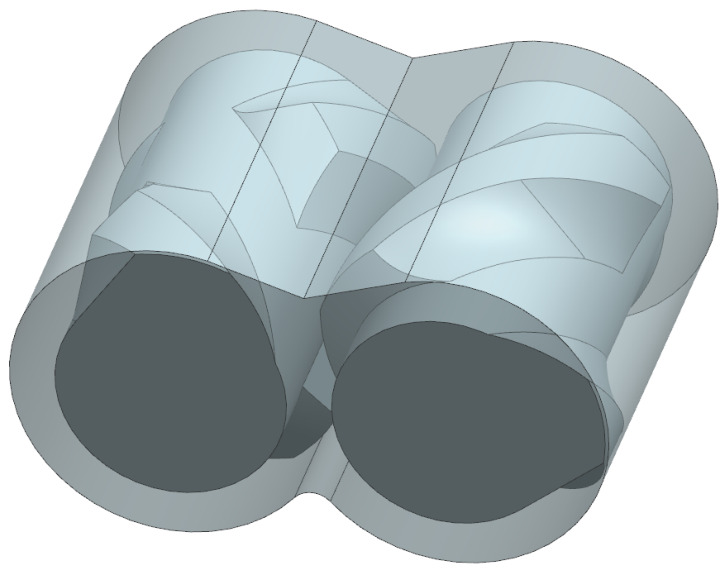
Model of internal mixer flow domain.

The internal mixer considered in this study consists of two counter-rotating rotors within an enclosed mixing chamber. The rotors operate at same speeds but in opposite directions to ensure the precise intermeshing of the rotor teeth. The modeled system represents an industrial-scale mixer with a total chamber volume of 0.4233 m^3^, a rotor length of approximately 1 m and a barrel diameter of approximately 0.5 m. The intermeshing rotor configuration creates narrow clearance regions between the rotor surfaces and the chamber walls, which are critical for generating high shear rates and extensional deformation during operation. The overall geometry is therefore representative of industrial-scale intermeshing mixers, although specific proprietary design details have been intentionally omitted for confidentiality. Figure 1 depicts the geometry.

### 2.2. Governing Equations and Constitutive Model

In this study, the rubber compound was modeled as an incompressible, isothermal, homogeneous non-Newtonian fluid. This assumption represents the compound as a single effective continuum during the dispersion and distribution stages of mixing. The effects of viscous heating, chemical reactions, filler network evolution, and explicit viscoelastic stress storage were not included, allowing the analysis to focus specifically on how operating parameters influence flow behavior and power consumption.

The homogeneous single-phase representation also neglects filler network evolution, bound rubber effects, and local polymer–filler interactions. These microstructural effects can influence the effective viscosity and energy dissipation behavior of filled rubber compounds; therefore, the present results should be interpreted as macroscopic continuum-level predictions rather than a full microstructural description of rubber mixing.

In practical mixing processes, temperature rise due to viscous dissipation can reduce material viscosity and consequently influence the absolute magnitude of power consumption. However, the present isothermal formulation provides a controlled framework for isolating the effects of operating parameters. While non-isothermal conditions may alter the magnitude of energy demand, the qualitative trends associated with fill factor, rotor speed, and rotor wear are expected to remain consistent, as they are primarily governed by geometric confinement and rotor kinematics.

Based on these assumptions, the governing equations for the flow field are given as follows.

(1) ∇⋅u=0

(2) $$\rho \left(\frac{\partial u}{\partial t}+u\cdot \nabla u\right)=-\nabla p+\nabla \cdot \tau +\rho g$$

Here, u is the velocity vector, ***t*** is time, ***p*** defines pressure, ρ is the fluid density, **g** is acceleration due to gravity and **τ** denotes the stress tensor. The stress tensor is defined as: $\tau =2\mu (\dot{\gamma })D$, where $\mu (\dot{\gamma })$ is the shear-rate-dependent viscosity, and the strain-rate tensor is denoted by **D**.

$D=\frac{1}{2}\left[\nabla u+{\left(\nabla u\right)}^{T}\right]$, where (.)^T^ denotes the transpose of the tensor.

The scalar shear rate is defined as: $\dot{\gamma }=\sqrt{2D:D}$.

Considering the non-Newtonian nature of the rubber, the viscosity μ is the function of local shear rate approximated using the Carreau–Yasuda Model [18] as:

(3) $$\mu (\dot{\gamma })=\mu_{\infty }+(\mu_{0}-\mu_{\infty }){\left[1+{\left(\lambda \dot{\gamma }\right)}^{a}\right]}^{\frac{n-1}{a}}$$

where $\mu_{\infty }$ is the infinite shear viscosity, $\mu_{0}$ is the zero-shear viscosity, $\dot{\gamma }$ is the local shear rate, λ is the relaxation time, n is the power law index and a is the flow behavior index.

The mixing chamber is partially filled, meaning that there is a need to track the rubber–air interface. The Euler-based VOF model [19] was used. The VOF model used is as follows:

(4) $$\frac{\partial \alpha }{\partial t}+\nabla \cdot (\alpha u)=0,\;0\leq \alpha \leq 1$$

where **α** is the volume fraction of the rubber phase, ***t*** is time, and **u** is the velocity vector. A value of α = 1 indicates a cell fully occupied by rubber, α = 0 indicates a cell occupied by air, and 0 < α < 1 represents the rubber–air interface. The operator ∇ denotes the gradient operator and ∇⋅represents divergence.

### 2.3. Mesh Independence Analysis

To ensure the numerical accuracy and reliability of the simulation results, a mesh independence study was conducted under representative operating conditions (fill factor = 65%, rotor speed = 50 rpm, unworn rotor).

**Figure 2:**
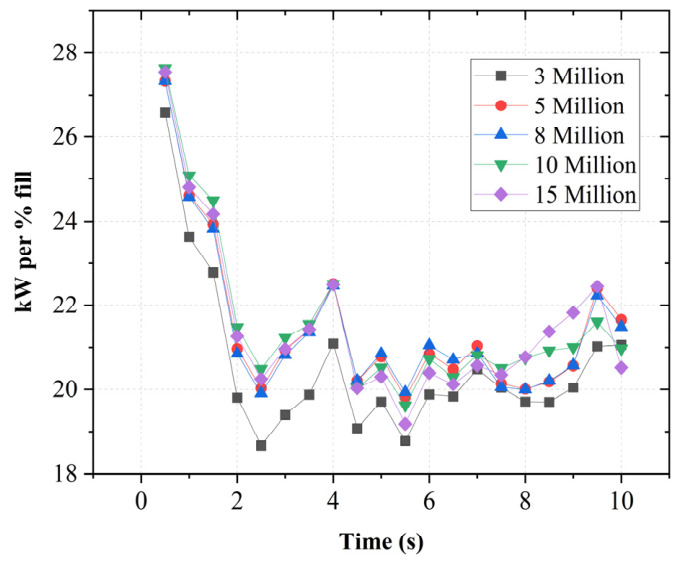
Mesh independence study.

Five levels of mesh resolution were examined, corresponding to approximately 3, 5, 8, 10, and 15 million cells. The total power consumption was used as the primary comparison metric as shown in Figure 2. While the coarsest mesh (3 million cells) underpredicts the power due to insufficient resolution of the intermeshing region, the results for 5 million cells and above show strong agreement. In particular, the differences between the 8, 10, and 15 million meshes remain within approximately 1–2%. The 5-million-mesh cell also falls within a similarly small deviation range.

Based on these results, a mesh resolution of approximately 5 million cells was selected for subsequent simulations, as it provides a good balance between numerical accuracy and computational cost, while remaining in close agreement with higher-resolution meshes.

### 2.4. Time-Step Independence Analysis

A time-step independence study was conducted to assess the sensitivity of the simulation results to temporal resolution under the same representative operating conditions (fill factor = 65%, rotor speed = 50 rpm, unworn rotor).

**Figure 3:**
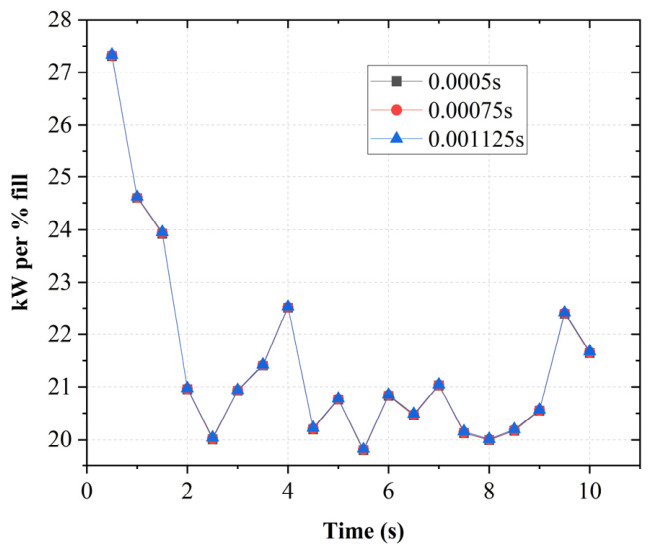
Time-step independence study.

Three time-step sizes (0.0005 s, 0.00075 s, and 0.001125 s) were evaluated. The results, shown in Figure 3, indicate that the predicted power consumption curves are nearly identical across all the selected time steps. The deviations are approximately 1%, indicating temporal convergence. However, larger time steps were observed to increase mesh distortion during dynamic remeshing, particularly in regions of close rotor clearance, leading to reduced mesh quality. Therefore, a time step of 0.00075 s was selected as an optimal compromise between numerical accuracy, mesh stability, and computational efficiency.

### 2.5. Model Mesh Information and Numerical Setup

**Figure 4:**
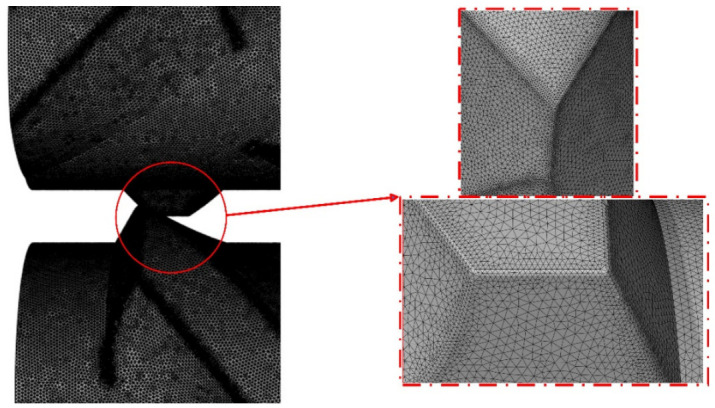
Tetrahedral mesh refinement in intermeshing zone of flow domain.

Numerical simulations were performed using the commercial CFD software ANSYS FLUENT version 2023 R2 under partially filled isothermal conditions. Due to the intermeshing nature of the rotors, a 3D dynamic remeshing method was employed to handle the constantly changing flow fields within the mixing chamber. Figure 4 below depicts the mesh of the rotors in the flow domain, highlighting the mesh refinements in the areas of interest.

A tetrahedral mesh of approximately 5 million elements was employed in the remeshing regions due to its flexibility in accommodating large deformations. During mesh refinement, the maximum cell skewness was targeted to remain below 0.7, and the minimum orthogonal quality above 0.35, ensuring that distortions did not severely degrade element quality. Thresholds for maximum cell skewness and maximum face skewness were defined so that elements were automatically re-meshed once these limits were reached, albeit at increased computational cost. The appropriate dynamic zones were assigned to ensure that areas such as the chamber wall which does not need remeshing would not re-mesh. This approach allowed accurate tracking of rotor–material interactions and avoided mesh collapse during close-clearance motion. The pressure–velocity coupling was performed using the PISO algorithm. Spatial gradients were computed using the least-squares cell-based method. Pressure interpolation was carried out using the PRESTO scheme to improve accuracy in regions with strong pressure variations. The momentum equations were discretized using a second-order upwind scheme to enhance solution accuracy. A first-order implicit transient formulation was adopted to ensure numerical stability during simulations involving dynamic remeshing and highly nonlinear flow behavior. The boundary conditions employed in the calculations are outlined in Table 1, while the rheological properties of the rubber are outlined in Table 2.

The rheological properties of the rubber compound were measured using a Rubber Process Analyzer (RPA-8000A, GOTECH Testing Machines Inc., Dongguan, China) under controlled oscillatory deformation. The instrument is compliant with GB/T 16584 [20] and ISO 6502 [21], which specify curemeter-based methods for evaluating the vulcanization and viscoelastic characteristics of rubber compounds. The measured viscoelastic response was used to derive the rheological parameters required for the non-Newtonian material model. Frequency sweep tests were conducted over a range of frequencies (0.1–20 Hz) and temperatures (40–170 °C), from which complex modulus (G^∗^) and complex viscosity ($\mu^{*}$) were determined.

Since RPA measurements provide oscillatory viscoelastic properties rather than steady shear viscosity, a transformation was applied to obtain an effective shear-rate-dependent viscosity suitable for CFD simulations. Specifically, the complex viscosity was related to angular frequency (ω=2πf) and interpreted in terms of an equivalent shear rate using the Cox–Merz-type approximation:

(5) $$\mu (\dot{\gamma })\approx \mu^{*}(\omega ),\;\dot{\gamma }=\omega$$

where $\mu (\dot{\gamma })$ is the shear-rate-dependent viscosity, $\mu^{*}(\omega )$ is the complex viscosity obtained from oscillatory measurements, $\dot{\gamma }$ is the shear rate, and ω is the angular frequency. This approach allows oscillatory rheological data to be mapped to an apparent steady shear viscosity representation. The resulting viscosity curve was then fitted using the well-known Carreau–Yasuda model [10,22,23] to obtain the relevant parameters $\mu_{0}$ and $\mu_{\infty }$, ensuring that the model captures the observed shear-thinning behavior across the relevant range of deformation rates. The estimation for relaxation time (λ) was achieved using the Weissenberg criterion [24], $\mathrm{Wi}\equiv {\dot{\gamma }}_{0}\lambda \approx 1$, hence $\lambda \approx \frac{1}{{\dot{\gamma }}_{0}}$.

The power law index (n) value was selected from literature [22,25], where it describes the shear-thinning behavior of rubber. While this approach does not fully account for viscoelastic effects such as elastic energy storage and stress relaxation, it provides a practical and widely used approximation for representing flow resistance in highly viscous polymer systems under high-shear conditions.

### 2.6. Case Matrix

**Figure 5:**
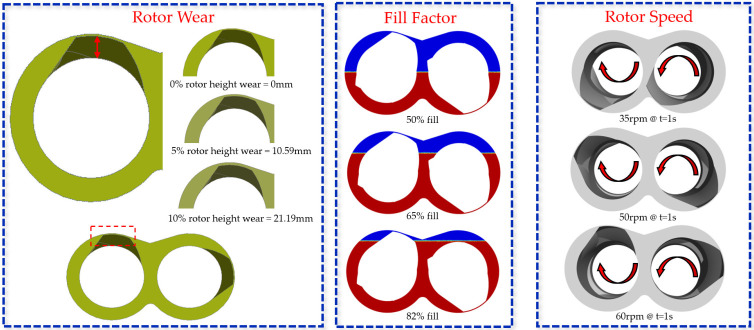
Various operating conditions.

A total of 7 cases were calculated across several operating conditions. Figure 5 shows the various conditions varied across the study. The fill factor was varied from 50% to 82%, where 82% presented the highest possible fill factor based on the design of the mixer. The rotor speeds were also varied from 35 rpm up to 60 rpm. Finally, the phenomenon of rotor wear was simulated by modifying the rotor height. Three wear states were chosen where 0% represents no wear (baseline geometry) and 5% wear depicts 95% of the total height of the rotor blades measured from the base of the rotor barrel to the tip. And finally, 10% wear represents 90% of the total height of the rotor. Table 3 provides a summary of the simulated operating conditions structured around a baseline condition (65% fill factor, 50 rpm, and 0% rotor wear). The individual parameters were varied independently to isolate their effects.

### 2.7. CFD Validation

**Figure 6:**
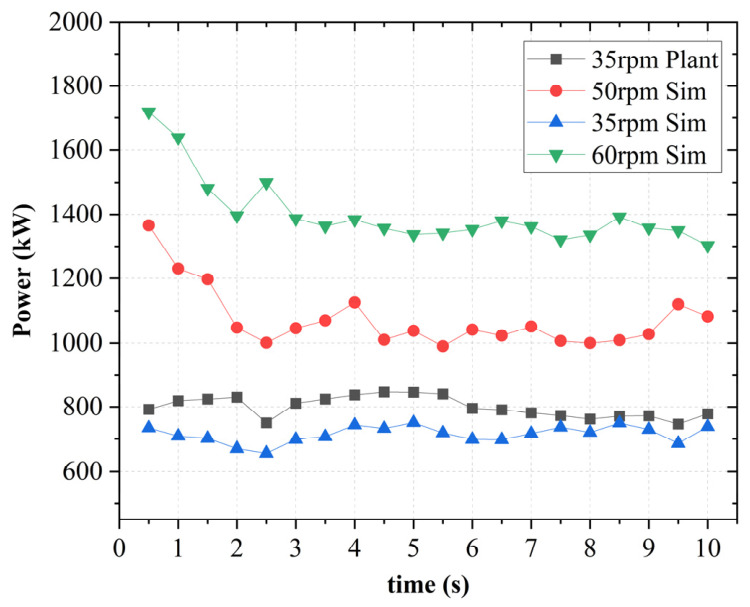
Performance characteristics of operational plant data against simulation.

Figure 6 depicts the performance characteristics of operational plant data against the simulation. Power consumption data were obtained from the factory’s intermeshing internal mixer during routine operation via the plant’s centralized monitoring system. Detailed information on sensor specifications and calibration was not available; therefore, a formal uncertainty analysis could not be performed.

Power consumption data were available only for the 35 rpm condition. Accordingly, validation is limited to this operating point. This section assesses whether the model reproduces the observed power consumption trends under representative industrial conditions. While some deviations in magnitude are observed, the overall agreement in trend and scale indicates that the model reasonably represents the dominant flow resistance and energy dissipation mechanisms governing power consumption in the mixer under the studied condition.

To quantify the agreement, the root mean square error (RMSE) and mean absolute percentage error (MAPE) are calculated as:

(6) $$RMSE=\sqrt{\frac{1}{n}\sum_{i=1}^{n}{\left(P_{sim,i}-P_{exp,i}\right)}^{2}}$$

(7) $$MAPE=\frac{1}{n}\sum_{i=1}^{n}\left|\frac{P_{sim,i}-P_{exp,i}}{P_{exp,i}}\right|\times 100$$

where $P_{sim,i}$ and $P_{exp,i}$ represent the simulated and experimental power values at time step *i* respectively, and *n* is the number of data points.

For the 35 rpm case, the RMSE was found to be 74.6 kW, while the MAPE was 8.4%. When normalized by the mean experimental power, this corresponds to a normalized RMSE (NRMSE) of approximately 9.4%. These values indicate that the model captures both the temporal evolution and magnitude of power consumption with reasonable accuracy for the available operating condition.

Based on this agreement at 35 rpm, the validated numerical framework is subsequently used to investigate the influence of other operating conditions, including higher rotor speeds (50 and 60 rpm), varying fill factors, and rotor wear. These additional cases should therefore be interpreted as model-based parametric predictions within the same computational framework.

## 3. Results

The ways in which various operating conditions affect the power consumption of the internal rubber mixer were evaluated. Seven numerical simulations of various fill factors, rotational speeds and rotor wear states were conducted for isothermal mixing of rubber. All simulations in this study were performed to represent the dispersion and distribution stages of rubber mixing. At this stage, it is assumed that all filler agglomerates have been fully incorporated into the rubber matrix, allowing the material to be treated as a single, continuous, highly viscous non-Newtonian fluid. Under these conditions, the focus is on the power drawn during redistribution and fine dispersion of components rather than initial incorporation. A complete analysis and discussion about the effect of different operating conditions on power consumption is presented here.

### 3.1. Rubber-Phase VOF

**Figure 7:**
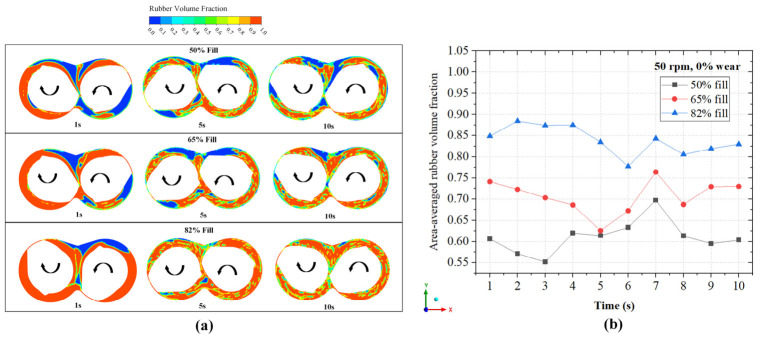
(**a**) Evolution of the rubber volume fraction at 1 s, 5 s, and 10 s for three fill factors (50%, 65%, and 82%) at 50 rpm and 0% rotor wear. (**b**) Area-averaged rubber volume fraction extracted from the same axial mid-plane shown in (**a**).

Figure 7a illustrates the evolution of the rubber volume fraction at 1 s, 5 s, and 10 s for three fill factors (50%, 65%, and 82%). For t = 1 s, for all fill levels, there is clear segregation and clearly defined rubber–air interfaces extending into the inter-rotor zone. It is also observed that the air pockets form at the trailing ends of the rotors. These air pockets are progressively smaller as fill factor increases. By time t = 5 s, there are relatively more air pockets dispersed within the rubber material. At t = 10 s, there appears to be a significantly dispersed concentration of air pockets within the rubber phase across all fill factors. However, the 50% fill case contains substantial air pockets, which reduce rubber occupancy near the rotor wall and inter-rotor zones and therefore limit the extent of material exposed to the high-shear-stress region. The opposite is true for the 82% fill factor. To quantify these trends, the area-averaged rubber volume fraction was extracted from the same axial mid-plane used for the contour plots, as shown in Figure 7b. An important feature of the quantified VOF response is that the mid-plane rubber occupancy does not vary only as a direct function of nominal fill level. For example, at t = 5 s, the 65% fill case shows a reduced area-averaged rubber volume fraction that approaches the 50% fill case. This indicates transient redistribution of the rubber–air interface through the sampled mid-plane rather than a simple monotonic dependence on the fill factor at every instant. This is in line with studies by Wang [10]. Nevertheless, the overall trend remains physically similar. The 82% fill case maintains the highest rubber volume fraction throughout the analyzed period, while the 50% fill case generally retains the lowest rubber volume fraction.

### 3.2. Influence of Fill Factor on Power Consumption

**Figure 8:**
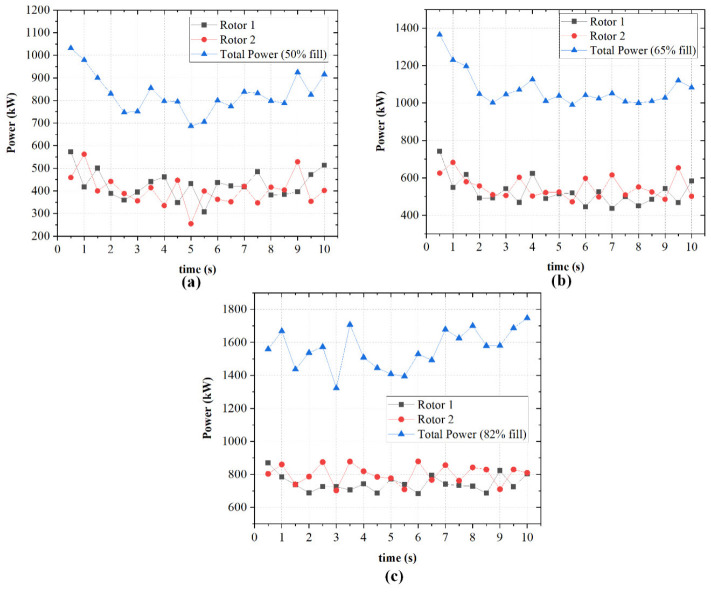
Temporal variation in power draw influenced by fill factor.

**Figure 9:**
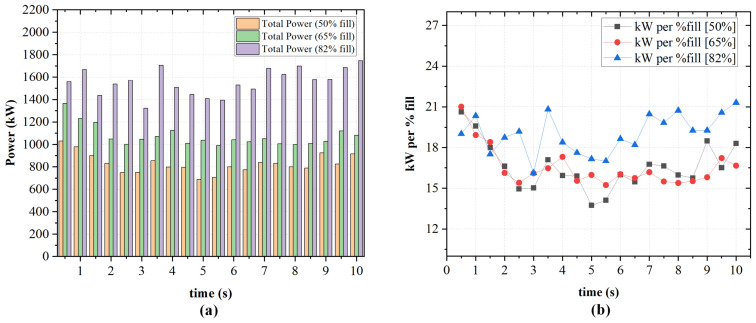
(**a**) Total power consumption for different fill factors (50%, 65%, and 82%) at 50 rpm and 0% rotor wear. (**b**) Corresponding normalized power consumption (kW per % fill) as a function of time.

Figure 8 presents the temporal variation in power draw for each rotor and the corresponding total power at three fill factors (50%, 65%, and 82%). As depicted in Figure 8a, at 50% fill, the rotors operate at comparatively low loads, drawing between approximately 250–550 kW each. The total power peaks near 1050 kW immediately after startup, then drops to about 750 kW within the first 3 s before stabilizing. This corresponds to normalized values of 14–19 kW/% fill as depicted in Figure 9b, the lowest across all cases. Increasing the fill factor to 65% as shown in Figure 6 raises rotor loads to a range of 450–650 kW. The total power starts around 1400 kW, decreases to 1040 kW after 3 s, and stabilizes thereafter. The normalized power also rises to 15–18 kW/% fill. At 82% fill, both rotors draw approximately 700–900 kW, the total power peaks near 1750 kW, and remains elevated throughout the simulation. This can be seen in Figure 8c. The normalized power in Figure 9b reaches 17–22 kW/% fill, indicating higher instantaneous torque demand. The non-linear increase in both absolute and normalized power from 65% to 82% fill is associated with increased viscous resistance under higher chamber occupancy. As the available free volume decreases, material circulation becomes more constrained, which corresponds to increased flow resistance and higher mechanical energy dissipation. This phenomenon is consistent with previous studies [22,26], which report that increasing the fill factor leads to increased flow resistance. This in turn increases mechanical loading due to reduced free volume within the mixing chamber.

In summary, increasing the fill factor from 50% to 65% results in a moderate increase in total power demand and torque loading that remains energetically efficient. However, further increasing the fill factor to 82% produces a substantial and sustained rise in both absolute and normalized power consumption. This is indicative of operational inefficiency and elevated mechanical loading consistent with increased flow confinement. A fill factor of 70 or 75% can also be considered in the future to study the trade-offs between fill efficiency and power consumption.

### 3.3. Influence of Rotor Speed on Power Consumption

**Figure 10:**
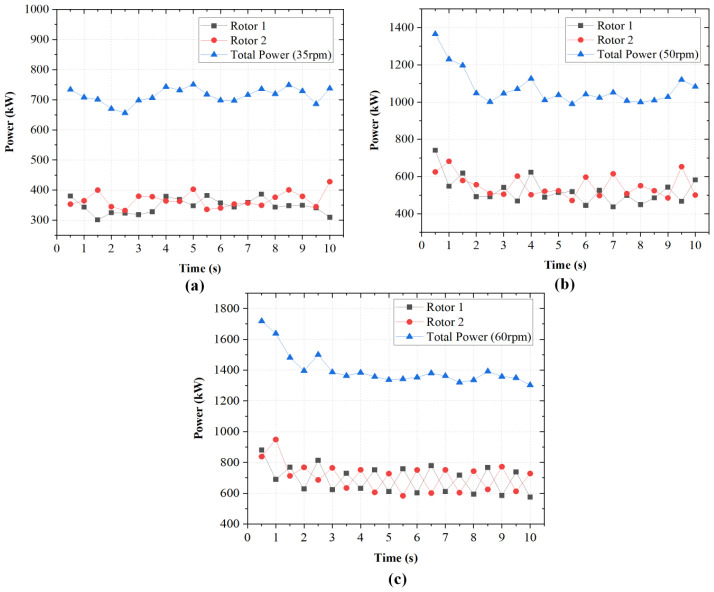
Temporal variation in power draw influenced by rotor speed.

**Figure 11:**
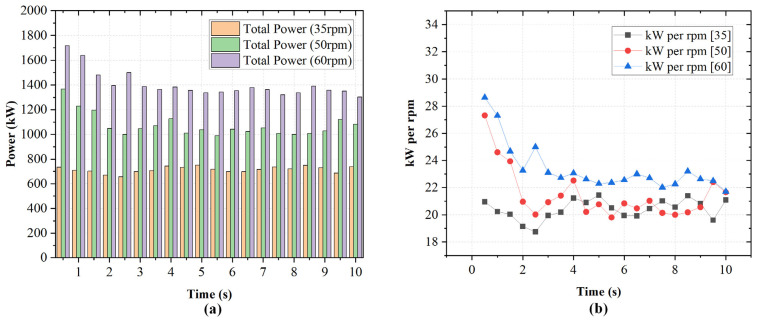
(**a**) Total power consumption for different rotor speeds (35 rpm, 50 rpm, and 60 rpm) at 65% fill factor and 0% rotor wear. (**b**) Corresponding normalized power consumption (kW per rpm) as a function of time.

Figure 10 shows the temporal variation in power draw for each rotor and the total power at three rotational speeds (35, 50, and 60 rpm). At 35 rpm, depicted by Figure 10a, the rotors draw 300–400 kW and 350–450 kW respectively. The total power peaks just above 750 kW during startup, before stabilizing between 650 and 750 kW. The normalized power remains lowest at 20–22 kW/rpm as depicted in Figure 11b. Increasing speed to 50 rpm as seen in Figure 10b raises rotor loads to 450–650 kW. The total power starts at 1400 kW, drops to 1050–1150 kW within 4 s, and stabilizes. The corresponding normalized values rise slightly to 20–23 kW/rpm. At 60 rpm, rotor loads increase substantially to 600–800 kW, with total power exceeding 1700 kW immediately after startup. This stabilizes after t = 3 s near 1400 kW. The normalized power reaches 22–23 kW/rpm. The non-linear increase in both absolute and normalized power with rotor speed suggests that power consumption becomes increasingly sensitive to rotational speed under the studied conditions. Similar trends have been reported in numerical studies of internal mixers [27,28], where increased rotational speed enhances shear rates and consequently increases energy dissipation and power demand.

Increasing rotor speed from 35 to 50 rpm results in a significant rise in total power consumption. Further increase to 60 rpm elevates the shear rate field within the chamber, indicating more intense material deformation and contributing to higher wall shear stress and torque. However, this improvement is accompanied by higher power consumption, indicating that rotor speed must be selected to balance mixing performance against energy demand. Further analysis is required to assess how these competing effects influence overall process performance.

### 3.4. Influence of Rotor Wear on Power Consumption

**Figure 12:**
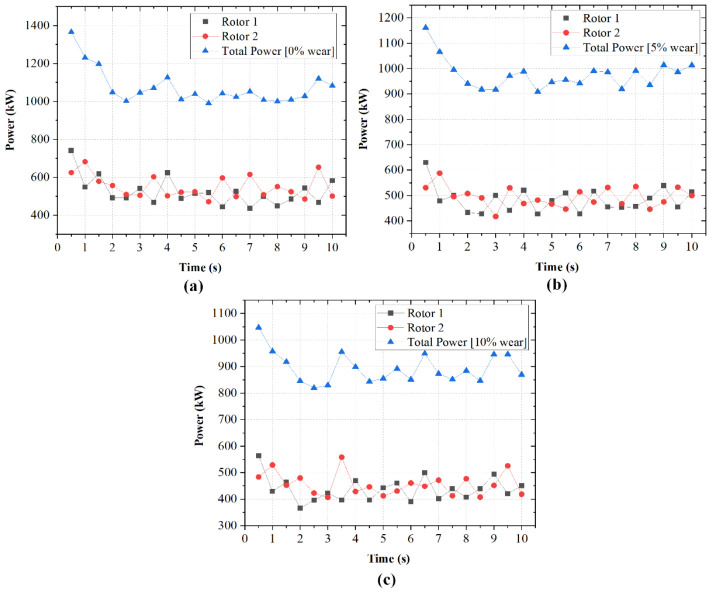
Temporal variation in power draw influenced by rotor wear.

The impact of rotor wear on instantaneous and total power consumption was evaluated for three wear levels: 0%, 5%, and 10% of the total rotor height. Figure 12 presents the temporal evolution of power draw for each rotor and the corresponding total power at each wear level.

**Figure 13:**
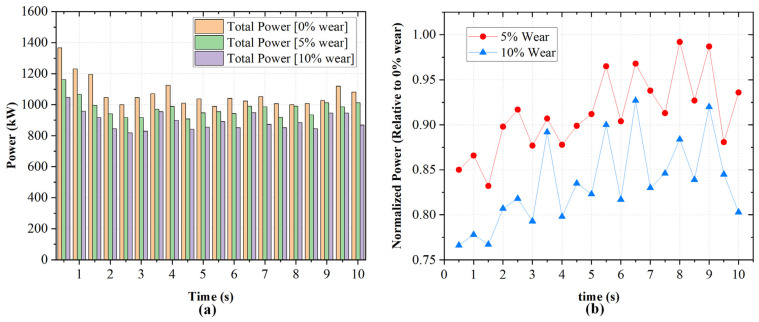
(**a**) Total power consumption for different wear states (0%, 5%, and 10%) at 50 rpm and 65% fill factor. (**b**) Corresponding normalized power relative to the unworn rotor (0% wear) as a function of time.

In Figure 12a, with unworn rotors, rotor loads are 500–600 kW each. The total power peaks just under 1400 kW before settling at 1000–1100 kW. In Figure 12b, at 5% wear, rotor loads decrease modestly, with total power stabilizing at 900–1050 kW. In Figure 12c, at 10% wear, loads are further reduced and total power remains 800–1000 kW. The progressive reduction in power with increasing wear reflects reduced rotor–material engagement. This diminishes shear stress generation due to increased clearances. To further quantify this effect, the power values were normalized with respect to the unworn rotors. The normalized power trends are shown in Figure 13b. The 5% wear condition retains approximately [~0.85–0.97] of the baseline rotor power consumption, while the 10% wear condition retains approximately [~0.77–0.92], depending on the transient rubber distribution within the chamber. This demonstrates that rotor wear progressively reduces the ability of the rotor to transfer mechanical energy to the rubber phase.

Although rotor wear has received limited attention in numerical studies, the observed reduction in power consumption with increasing wear can be attributed to increased effective flow volume and reduced shear stress intensity, consistent with general principles of viscous dissipation in confined flows.

### 3.5. Power Density Normalization

To enable geometric scaling of the reported power values, the power in the steady operating region was normalized by the chamber volume to obtain power density (kW/m^3^). The steady-state values were determined by averaging the total power over the interval t = 3–10 s.

For the fill factor and rotor speed cases, a constant chamber volume of 0.4233  m^3^ was used. For the rotor wear cases, the power density was calculated using the corresponding chamber volume for each worn-rotor geometry. This is because rotor wear modifies the effective flow domain by increasing internal clearances.

The resulting steady-state power and corresponding power density values for all simulated operating conditions are summarized in Table 4.

### 3.6. Influence of Operating Conditions on Wall Shear Stress

Wall shear stress is a primary contributor to torque generation and mechanical power input in internal mixers, and is therefore analyzed as a key parameter for characterizing rotor–material interaction. Shear stress at the rotor–material interface represents the direct physical mechanism through which the rotors impart work to the rubber compound. Higher wall shear stress magnitudes and broader coverage typically indicate increased viscous resistance, translating into greater torque demand.

**Figure 14:**
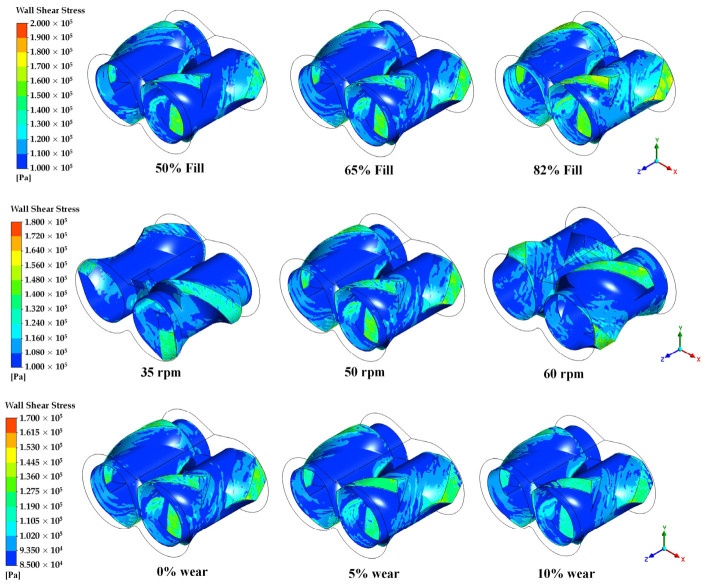
Influence of operating conditions on wall shear stress.

It is observed in Figure 14 that wall shear stress increases with the fill factor both in magnitude and spatial coverage. This indicates more extensive and sustained rotor–material contact under higher load conditions. However, the elevated shear stress at high fill levels also implies increased torque demand. Also, wall shear stress increases non-linearly with rotor speed, reflecting the combined influence of higher relative velocity and increased material acceleration on stress generation at the rotor–material interface. Finally, the simulation data indicates that rotor wear leads to a measurable reduction in wall shear stress magnitude and distribution, consistent with increased clearances and reduced mechanical engagement of the rotor surfaces with the bulk material.

Table 5 outlines the comparative effects of operating parameters on power consumption and wall shear. It summarizes the comparative effects of the operating parameters on normalized power consumption and shear coverage within the mixer. The final column provides a physical interpretation of how each operating condition influences flow behavior and power consumption.

## 4. Conclusions

This study employed a validated three-dimensional CFD model with dynamic remeshing to quantify how fill factor, rotor speed, and rotor wear influence the power consumption of an isothermal intermeshing internal mixer. The analysis, supported by wall shear stress distributions, provides insight into the flow features governing energy demand under the conditions studied.

Increasing the fill factor from 50% to 65% resulted in a moderate rise in power consumption while maintaining relatively stable normalized power values (15–18 kW/% fill). Further increasing the fill factor to 82% led to a disproportionate increase in both absolute and normalized power (17–22 kW/% fill), consistent with increased flow confinement and viscous resistance, as reflected by the expanded high-shear-stress regions observed in wall shear contours.

Increasing rotor speed from 35 rpm to 50 rpm significantly increased torque and power consumption, while operation at 60 rpm produced a non-linear rise in energy demand (22–23 kW/rpm). Wall shear stress analysis showed more concentrated high-shear-stress regions at higher speeds, which is consistent with the observed increase in torque.

Progressive rotor wear from 0% to 10% reduced both absolute and normalized power consumption due to decreased rotor–material engagement and reduced shear stress generation. The corresponding reduction in high-shear-stress coverage may indicate reduced high-intensity deformation, which could influence dispersive processes.

Within the range of conditions investigated, intermediate operating conditions (e.g., 65% fill factor and 50 rpm) exhibited a balance between increased power demand and shear stress generation, relative to lower and higher operating extremes. These results highlight the trade-offs between mechanical loading and flow intensity in intermeshing mixers.

By linking power consumption to wall shear stress behavior, this study provides a mechanistic framework for interpreting how operating conditions influence energy demand and flow characteristics in internal mixing. The methodology can be extended to non-isothermal conditions and additional parameters to further explore process behavior in industrial rubber mixing systems.

It should be noted that the present study assumes isothermal conditions. In practical systems, temperature rise during mixing may reduce viscosity and consequently lower absolute power consumption. However, the observed qualitative trends with respect to fill factor, rotor speed, and rotor wear are expected to persist, as they are primarily governed by flow confinement, rotor kinematics, and geometric interactions. Future work incorporating temperature-dependent rheology and energy coupling would provide further insight into these effects.

## Figures and Tables

**Table 1:** Boundary conditions.

Boundary Conditions	Settings
Turbulence Model	None (Laminar Flow)
Interface Configuration	Transient Rotor–Stator
Transient Timestep Size	0.00075 s
Total Run Time	10 s
Wall Roughness	Smooth Wall
Shear Condition	No Slip
Numerical Scheme	First-Order Implicit
Convergence Criterion	10^−6^

**Table 2:** Rheological properties of rubber.

Property	Value
Zero-Shear Viscosity ($\mu_{0}$)	1,107,000 Pa.s
Infinite-Shear Viscosity ($\mu_{\infty }$)	13,000 Pa.s
Relaxation Time (λ)	5 s
Power Law Index (n)	0.15
Rubber Density (ρ)	1100 kg/m^3^

**Table 3:** Summary of simulated operating conditions.

Case	Fill Factor (%)	Rotor Speed (rpm)	Rotor Wear (%)	Description
C1	65	50	0	Baseline Case
C2	50	50	0	Fill Factor Variation
C3	82	50	0	Fill Factor Variation
C4	65	35	0	Speed Variation
C5	65	60	0	Speed Variation
C6	65	50	5	Wear Variation
C7	65	50	10	Wear Variation

**Table 4:** Steady-state power and power density for all operating conditions.

Parameter	Condition	Chamber Volume (m^3^)	Approx Steady Total Power, kW	Power Density, kW/m^3^
Fill factor	50%	0.4233	805.94	1903.95
	65%	0.4233	1043.21	2464.47
	82%	0.4233	1560.63	3686.82
Rotor speed	35 rpm	0.4233	721.15	1703.64
	50 rpm	0.4233	1043.21	2464.47
	60 rpm	0.4233	1355	3201.04
Rotor wear	0% wear (100% rotor height)	0.4233	1043.21	2464.47
	5% wear (95% rotor height)	0.4267	964.71	2260.86
	10% wear (90% rotor height)	0.4301	886.17	2060.38

**Table 5:** Comparative effects of operating parameters on power consumption and wall shear stress.

Parameter	Condition	Normalized Power	Shear Stress Coverage	Physical Interpretation
Fill factor	50%	14–19 kW/% Fill	Manifests in localized regions, mostly on the rotor tips	Lower chamber occupancy reduces flow resistance and power demand, with higher shear stress localized near rotor tips.
	65%	15–18 kW/% Fill	Shear stress coverage is broader compared to 50% fill	Intermediate fill level corresponds to more distributed shear coverage with moderate power demand.
	82%	17–22 kW/% Fill	Shear stress coverage is extensive, to the rotor barrels.	Higher fill level increases flow confinement, associated with elevated torque and shear stress levels.
Rotor speed	35 rpm	20–22 kW/rpm	Limited coverage. In localized regions, mostly on the rotor tips	Lower rotational speed corresponds to reduced shear stress intensity and lower energy input.
	50 rpm	20–23 kW/rpm	Broader coverage, with higher shear stress on the rotor tips	Moderate rotational speed increases shear stress intensity with a corresponding rise in power demand
	60 rpm	22–23 kW/rpm	High-shear-stress coverage is comparable to the 50 rpm case, with increased localization of shear near the rotor tips	Higher rotational speed is associated with increased torque and wall shear stress, with a non-linear rise in energy demand.
Rotor wear	0% wear	1	Concentrated high-shear-stress zones, particularly along the leading faces and tips	Baseline geometry produces higher shear stress intensity due to minimal rotor–wall clearance.
	5% wear (95% rotor height)	0.85–0.936 (Relative to 0% wear)	High-shear zones become less intense and slightly reduced in spatial coverage	Reduced rotor height decreases shear stress intensity and slightly lowers power demand.
	10% wear (90% rotor height)	0.766–0.803 (Relative to 0% wear)	More wear further reduces both the magnitude and coverage of high-shear-stress zones	Increased rotor wear results in lower torque and shear stress levels and hence power use

## Data Availability

The data that support the findings of this study have been reviewed and approved for use by the research institution. Due to confidentiality agreements, the datasets are not publicly available, but they may be made available from the corresponding author on reasonable request, subject to institutional approval.

## Abbreviations

The following abbreviations are used in this manuscript:

CFD	Computational Fluid Dynamics
UDF	User-Defined Function
VOF	Volume of Fluid
MI	Mixing Index
**Nomenclature**	
*P*	pressure, Pa
*ρ*	density, kg/m^3^
*t*	time, s
*g*	gravity, m/s^2^
τ	stress tensor
*μ*	viscosity, Pa.s
*λ*	relaxation time, s
${\dot{\gamma }}_{0}$	strain-rate amplitude, s^−1^
